# Consensus statement for perioperative care in total hip replacement and total knee replacement surgery: Enhanced Recovery After Surgery (ERAS^®^) Society recommendations

**DOI:** 10.1080/17453674.2019.1683790

**Published:** 2019-10-30

**Authors:** Thomas W Wainwright, Mike Gill, David A McDonald, Robert G Middleton, Mike Reed, Opinder Sahota, Piers Yates, Olle Ljungqvist

**Affiliations:** a Orthopaedic Research Institute, Bournemouth Univesity, Bournemouth, UK;; b The Royal Bournemouth and Christchurch Hospitals NHS Foundation Trust, Bournemouth, UK;; c Golden Jubilee National Hospital, Glasgow, Scotland;; d Scottish Government, Glasgow, Scotland;; e Nursing, Midwifery and Allied Health Professions Research Unit, Glasgow Caledonian University, Glasgow, Scotland;; f Poole Hospital NHS Foundation Trust, Poole, UK;; g Northumbria Healthcare NHS Foundational Trust, Northumbria, UK;; h Health Sciences, University of York, York, UK;; i Nottingham University Hospital, Nottingham, UK;; j Nottingham University, Nottingham, UK;; k University of Western Australia, Perth, Australia;; l Örebro University, Örebro, Sweden

## Abstract

Background and purpose — There is a large volume of heterogeneous studies across all Enhanced Recovery After Surgery (ERAS^®^) components within total hip and total knee replacement surgery. This multidisciplinary consensus review summarizes the literature, and proposes recommendations for the perioperative care of patients undergoing total hip replacement and total knee replacement with an ERAS program.

Methods — Studies were selected with particular attention being paid to meta-analyses, randomized controlled trials, and large prospective cohort studies that evaluated the efficacy of individual items of the perioperative treatment pathway to expedite the achievement of discharge criteria. A consensus recommendation was reached by the group after critical appraisal of the literature.

Results — This consensus statement includes 17 topic areas. Best practice includes optimizing preoperative patient education, anesthetic technique, and transfusion strategy, in combination with an opioid-sparing multimodal analgesic approach and early mobilization. There is insufficient evidence to recommend that one surgical technique (type of approach, use of a minimally invasive technique, prosthesis choice, or use of computer-assisted surgery) over another will independently effect achievement of discharge criteria.

Interpretation — Based on the evidence available for each element of perioperative care pathways, the ERAS^®^ Society presents a comprehensive consensus review, for the perioperative care of patients undergoing total hip replacement and total knee replacement surgery within an ERAS^®^ program. This unified protocol should now be further evaluated in order to refine the protocol and verify the strength of these recommendations.

Over the last 15 years, the systematic implementation of an evidence-based perioperative care protocol (“fast-track” or “enhanced recovery pathway”), such as that developed by the Enhanced Recovery After Surgery (ERAS) Society, has shown that hospital length of stay and complications can be reduced for a number of surgical procedures (Ljungqvist et al. 2017). For total hip (THR) and total knee replacement surgery (TKR), high-volume models have reduced length of stay from 4–10 days to 1–3 days, and outpatient surgery is possible for around 15% of patients in unselected cohorts within a socialized health systems (den Hartog et al. 2013, Kehlet 2013, Khan et al. 2014, Aasvang et al. 2015, Gromov et al. 2017). Given the proven benefit to both the patient and the healthcare system, ERAS protocols have been published for rectal, urological, pancreatic, gastric, breast, and reconstructive surgery, head and neck cancer surgery, bariatric, and liver surgery (Cerantola et al. 2013, Nygren et al. 2012, Lassen et al. 2012, Melloul et al. 2016, Mortensen et al. 2014, Thorell et al. 2016, Dort et al. 2017, Temple-Oberle et al. 2017).

In Denmark, hip and knee replacement surgery has since year 2009 been coordinated nationally across select high-volume centers, adopting and developing a unified perioperative protocol, and a basis for research that has advanced the knowledge base for elective hip and knee replacement (Kehlet 2013). In the United Kingdom (UK), a national enhanced recovery partnership program ran between 2009 and 2011 with the aim to spread the best practice developed for hip and knee replacement based on work from UK high-volume centers (Wainwright and Middleton 2010, Malviya et al. 2011, McDonald et al. 2012). However, despite these efforts and previous narrative reviews on fast-track/enhanced recovery protocols for THR and TKR (Ibrahim et al. 2013a, 2013b; Sprowson et al. 2013), a systematic and evidence-based guideline has not been produced. The present work brought together a group of ERAS experts to interpret the evidence using the GRADE system for rating quality of evidence and strength of recommendations (Guyatt et al. 2008).

The scope of these guidelines includes only the perioperative period, with evidence examined on the ability of different interventions to reduce perioperative stress, maintain and support homeostasis and physiological function, and importantly accelerate the achievement of discharge criteria, including minimizing complications. Whilst the site of replacement is an independent factor for recovery, wherever possible we have prioritized procedure level evidence and applied critical analysis to studies that have included both THR and TKR.

This article represents the efforts of the ERAS Society (http://www.erassociety.org) to present an updated and expanded consensus review of perioperative care for hip and knee replacement based on current evidence.

## Methods

### Development of consensus recommendations

A panel of experts in total hip replacement and total knee replacement was convened. This working group comprised surgeons, a physician, an anesthetist, and physiotherapists. A nursing perspective was also incorporated through the consideration and inclusion of qualitative nursing-specific literature (Specht et al. 2015, 2016). Previous ERAS Society guidelines were reviewed and used as a methodological template (Gustafsson et al. 2013). The panel was asked to advise on appropriate topics to be included in the guidelines, with final decisions being made by the lead authors (TW, OL). Once agreed, topics were allocated to authors, depending on each individual’s expertise. The final paper was agreed upon by all authors.

### Literature search and study selection

Search terms were created using MESH terms and key words, and searches were carried out using the Bournemouth University MySearch interface (Bournemouth University), which includes Medline, CINAHL, Science Direct, PsycINFO, and Cochrane Database of Systematic Reviews databases. Whilst ERAS has evolved over the last 10–15 years, for completeness the peer-reviewed and English-language literature was systematically reviewed from January 1966 to October 2018. We appreciate that evidence from ERAS studies is evolving at a fast rate and so future studies may impact on findings; however, the recommendations presented are considered to reflect evidence at the time of writing (January 2019). Preoperative, surgical, anesthetic and analgesia, postoperative, and rehabilitation topics were searched. Reference lists of eligible articles were also reviewed for other relevant studies. Key words included “hip replacement,” “hip arthroplasty,” “knee replacement,” “knee arthroplasty,” “hip prosthesis,” “knee prosthesis,” and additional keywords were added depending on the topic.

The authors screened titles and abstracts to identify potentially relevant articles, and reference lists of eligible articles were hand-searched for relevant studies. Systematic reviews, meta-analyses, randomized controlled trials, and non-randomized trials were considered for each topic, unless there were few papers identified, in which case all papers were screened. Qualitative studies were also considered in specific areas, where “hands on” experience of ERAS was described. We consider that although these do not qualify as high-level evidence, they provide valuable evidence on issues such as how patients perceive the ERAS pathway, and the role that nurses play. We carefully reviewed the final literature selected, and any discrepancies were resolved through group consensus. Studies in ERAS or fast-track set-ups showing improvements in the achievement of discharge criteria, or reducing length of stay, or having a positive effect on complications were targeted.

### Quality assessment and data analyses

The overall quality of evidence was assessed using criteria developed by the Centre for Evidence Based Medicine at Oxford, England. Possible levels of evidence included “high” (i.e., systematic reviews, meta-analyses, or robust randomized controlled trials), “moderate” (i.e., smaller randomized controlled trials or prospective cohort data), or “low” (i.e., retrospective data). In line with ERAS guidelines for other surgical procedures (Gustafsson et al. 2013) the Grading of Recommendations, Assessment, Development and Evaluation (GRADE) system was used to evaluate the quality of evidence and recommendations (Tables 1 and 2, Guyatt et al. 2008). Recommendations are made based on whether the evidence level is high, medium, low, or very low quality, and the strength of the recommendation is based on the balance between desirable and undesirable effects. As with other ERAS working groups (Cerantola et al. 2013, Gustafsson et al. 2013), a GRADE evaluation may result in a strong recommendation even when based on low-quality data, if the risk of harm is negligible. Conversely, a weak recommendation may result from high-quality data. Any disagreements in the assessment of quality of evidence and grading of recommendation statements were resolved through consensus discussions. We were judicious when providing strong recommendations in areas where there was weak procedure-specific evidence. This was to ensure that new non-evidenced based traditions within ERAS were not created, when previous work in ERAS for THA and TKA has cautioned against this (Husted et al. 2014).

**Table 1.** GRADE system for rating quality of evidence (Guyatt et al. 2008)

Evidence level	Definition
High quality	Further research unlikely to change confidence in estimate of effect
Moderate quality	Further research likely to have important impact on confidence in estimate of effect and may change the estimate
Low quality	Further research very likely to have important impact on confidence in estimate of effect and likely to change the estimate
Very low quality	Any estimate of effect is very uncertain

**Table 2.** GRADE system for rating strength of recommendations (Guyatt et al. 2008)

Recommendation strength	Definition
Strong	When desirable effects of intervention clearly outweigh the undesirable effects, or clearly do not
Weak	When trade-offs are less certain—either because of low-quality evidence or because evidence suggests desirable and undesirable effects are closely balanced

## Evidence base and recommendations—ERAS items

### Preoperative information education and counselling

Preoperative patient education has not been shown to independently affect postoperative outcomes, such as accelerating the achievement of discharge criteria, but it has been found to reduce preoperative anxiety across a number of systematic reviews (Bergin et al. 2014, Jordan et al. 2014, Louw et al. 2013), including a Cochrane review (McDonald et al. 2014). However, the conclusions of these reviews may be flawed due to the heterogeneity of the pooled studies. There is a strong need for properly designed randomized and controlled studies that are sufficiently powered, performed in ERAS settings, and allow for discrimination between outcome parameters. Further information on what type of information should be given, at what point, by whom, and, for example, whether it should it be graded between younger active, older active, and older sedentary patients is required. Whilst strong specific evidence may be lacking to recommend preoperative education and counselling, qualitative studies detailing the patient perspective highlight the importance of patients getting the right information and support (Specht et al. 2016). Preoperative education is also unlikely to cause harm and is delivered in differing forms within all established hip and knee replacement ERAS centers. This means that it is a strongly recommended component.

***Summary/recommendation****—Preoperative patient education is recommended*

***Evidence level****—Low*

***Recommendation grade****—Strong*

### Preadmission patient optimization

Optimizing preoperative risk factors, such as smoking, alcohol consumption, anemia, nutritional and metabolic status, and low physical activity, that may lead to complications or a prolonged length of stay, could potentially benefit a large proportion of hip and knee replacement patients (Hansen et al. 2012). In the ERAS specific literature, preoperative screening and intervention has helped reduce the number of patients with delayed recovery (Hansen et al. 2012). The effect of specific individual factors on ERAS outcomes is considered below.

### Smoking cessation

The risk of an increased length of stay and other early postoperative complications for patients who smoke has been found to be reduced within established ERAS pathways (Jorgensen and Kehlet 2013). However, the association between current and former smoking and a substantially higher risk of postoperative complications and mortality post-surgery has level 1 evidence (Singh 2011). There is level 2 evidence across surgical studies to show that referral to a smoking cessation program 4 weeks preoperatively is associated with fewer complications, especially wound-related problems (Moller et al. 2002, Mills et al. 2011, Mak et al. 2014, Thomsen et al. 2014).

***Summary/recommendation****—4 weeks or more smoking cessation is recommended before hip and knee replacement*

***Evidence level****—High*

***Recommendation grade****—Strong*

### Alcohol

A large retrospective study found that hip and knee replacement patients who misused alcohol had a longer length of stay and were more likely to have medical and surgery related complications (Best et al. 2015). However, whilst this risk of a longer length of stay and other early postoperative complications may be less for patients on exemplar hip and knee replacement ERAS pathways (Jorgensen and Kehlet 2013), in line with evidence from other surgical procedures and public health recommendations, alcohol cessation interventions before surgery should be utilized to reduce complications in patients with high alcohol intake (Oppedal et al. 2012).

***Summary/recommendation****—Alcohol cessation programs are recommended before hip and knee replacement*

***Evidence level****—Low*

***Recommendation grade****—Strong*

### Anemia

Preoperative anemia is associated with an increased risk of transfusion, length of stay, infection, morbidity, and readmission rates (Kehlet 2013, Munoz et al. 2014), with the prevalence in elective hip and knee surgery reported as ranging from 15–39% (Spahn 2010).

RCTs and cohort studies of interventions such as preoperative iron or erythropoietin therapy and postoperative re-transfusion of salvaged cells in general report a statistically significant and clinically relevant reduction in allogeneic blood transfusion (Munoz et al. 2014, Theusinger el al. 2014). Algorithm-led preoperative anemia screening in established ERAS centers has also been associated with reduced RBC transfusion, readmission, critical care admission, length of stay, and costs (Pujol-Nicolas et al. 2017). The cause of the anemia must also be investigated and managed.

***Summary/recommendations****—Preoperative anemia should be identified, investigated, and corrected prior to hip and knee replacement.*

***Evidence level****—High*

***Recommendation grade****—Strong*

### Preoperative physiotherapy

Preoperative physiotherapy (including exercise programs) has been proposed as an intervention to expedite discharge (Carli and Zavorsky 2005). However, whilst preoperative physiotherapy may slightly improve early postoperative pain and function, the effects of the intervention in isolation remain too small to be considered clinically important and do not accelerate achievement of discharge criteria or shorten length of hospital stay (Wang et al. 2016). In addition, 1–2 day length of stay for unselected patients and outpatient surgery for hip and knee replacement has been achieved without preoperative physiotherapy (Gromov et al. 2017). Several reviews of RCTs of variable quality indicate little clinical benefit from preoperative physiotherapy (Wang et al. 2016, Moyer et al. 2017, Chen et al. 2018).

There is emerging work examining the addition of nutrition therapy and psychological preparation to exercise regimes, in a concept called prehabilitation. These programs have shown improvements to recovery in general surgical procedures (Carli and Scheede-Bergdahl 2015). However, procedure specific studies in hip and knee replacement are needed before recommendation, and the effect may only be seen in specific patient groups, such as elderly and frail patients, patients with special needs or multiple comorbidities, patients with psychiatric diseases, and patients not currently able to achieve discharge on the day of surgery (Bandholm et al. 2018).

***Summary/recommendation****—Current evidence does not support preoperative physiotherapy as an essential intervention*

***Evidence level****—Moderate (for not recommending)*

***Recommendation grade****—Strong*

### Preoperative fasting

Recent anesthetic guidelines indicate that the intake of clear fluids until 2 hours before surgery does not increase gastric content, reduce gastric fluid pH, or increase complication rates. Therefore, the intake of clear fluids until 2 hours before the induction of anesthesia as well as a 6-hour fast for solid food is recommended (Smith et al. 2011). To facilitate this, specific guidelines should be provided to each individual patient depending on their time of surgery and place on the operating list.

***Summary and recommendation****—Intake of clear fluids until 2 hours before the induction of anesthesia, and a 6-hour fast for solid food is recommended*

***Evidence level****—Moderate*

***Recommendation grade****—Strong*

### Preoperative carbohydrate treatment

Carbohydrate loading has been shown to reduce insulin resistance in various surgical procedures including orthopedic surgery (Nygren 2006, Awad et al. 2013). Meta-analysis data suggest shorter length of stay after major abdominal surgery but not in hip and knee replacement (Smith et al. 2014). In hip replacement, some small RCTs show positive effects on preoperative hunger and nausea, and postoperative pain (Harsten et al. 2012) as well as on glucose metabolism (Soop et al. 2004) and insulin resistance (Nygren et al. 1999), while others show no effect (Ljunggren and Hahn 2012). Whilst carbohydrate loading may improve patient well-being perioperatively (Harsten et al. 2012), outpatient surgery (Gromov et al. 2017) and routine 1–2-day length of stay on unselected patients (Aasvang et al. 2015) is achievable without carbohydrate loading. Therefore, the current evidence does not support the routine use of carbohydrate loading; however, future research may elicit benefits for more elderly and frail patients, and patients with multiple comorbidities.

***Summary/recommendation****—In hip and knee replacement carbohydrate loading may improve patient well-being and metabolism, but it has not been shown to accelerate the achievement of discharge criteria or reduce complications, and so it is not currently recommended as an essential routine intervention*

***Evidence level****—Moderate (for not recommending)*

***Recommendation grade****—Strong*

### Pre-anesthetic medication

Sedative or anxiolytic drugs may be used to promote patient comfort and/or facilitate the successful completion of technical procedures such as spinal anesthesia. However, their use is not universal and side effects may include postoperative sedation. There are minimal data available (for or against) to support the preoperative use of sedative or anxiolytic medication to reduce anxiety and accelerate the achievement of discharge criteria (Moiniche et al. 2002). If indicated, short-acting sedative drugs may be used by the clinician to facilitate successful completion of technical procedures, but routine administration of sedatives to reduce anxiety preoperatively should be avoided.

***Summary and recommendation****—The routine administration of sedatives to reduce anxiety preoperatively is not recommended*

***Evidence level****—Low*

***Recommendation grade****—Strong*

### Standardized anesthetic protocol

A core component of hip and knee replacement ERAS pathways is a standardized anesthetic protocol. However, the components of these protocols differ, and a lack of methodological quality, reporting detail, and homogeneity of outcome measures makes direct comparison of techniques in ERAS settings difficult (Kehlet and Aasvang 2015).

### The use of general versus central neuraxial anesthesia

In general, large multi-center cohorts of exemplar ERAS hip and knee replacement pathways favor neuraxial techniques over general anesthesia, and this change in practice has been at the core of established ERAS pathways (McDonald et al. 2012, Khan et al. 2014). Large epidemiological studies (albeit in non-ERAS set ups) show that central neuraxial anesthesia is independently associated with better outcomes compared with general anesthesia (Memtsoudis et al. 2013). Conversely, emerging research from 2 single-center RCTs in established ERAS centers has questioned whether the reduced cardiopulmonary and thromboembolic complications associated with neuraxial techniques (Harsten et al. 2013, 2015b are as relevant in an ERAS set up. A modern general anesthetic was compared with a traditional high dose of neuraxial anesthesia (bupivacaine 0.5% 3 mL), and no clinically relevant differences in functional recovery, length of stay, urinary complications, and mobilization were found. However, further studies are needed to compare modern general anesthetic and neuraxial anesthesia practices. General anesthesia may also reduce urinary bladder dysfunction, and rare but potentially severe neurological complications (Kehlet and Aasvang 2015). Future multi-center RCTs are required to further compare the safety issues and potential differences in postoperative morbidity between the 2 anesthetic techniques with specific emphasis on detailing the components of each technique, e.g., type of spinal.

***Summary and recommendations****—Modern general anesthesia and neuraxial techniques may both be used as part of multimodal anesthetic regimes*

***Evidence level****—Moderate (modern general anesthesia), moderate (neuraxial techniques)*

***Recommendation grade****—Strong*

### Spinal (intrathecal) opioids

There has been considerable interest in the use of opioids as an addition to local anesthetic in spinal anesthesia for hip and knee replacement. Whilst spinal opioids have been shown to lower pain scores and analgesic use (Cole et al. 2000), they increase the risk of urinary retention, pruritus, and respiratory depression (Gehling and Tryba 2009, Fernandez et al. 2014). Side effects may be avoided when lower doses are used, however, any superior effect on pain is then lost compared to alternative techniques such as local infiltrative analgesia (LIA) in knee replacement (Andersen and Kehlet 2014). Therefore, despite the analgesic benefits, the potential for unwanted side effects such as respiratory depression, postoperative nausea and vomiting, and pruritus does not support the routine use of spinal opioids.

***Summary and recommendations****—Spinal opioids are not recommended for routine use*

***Evidence level****—Moderate*

***Recommendation grade****—Strong*

### Epidurals

Lumbar epidurals have been accepted as providing favorable analgesia postoperatively for lower limb surgery. However, there are potential side effects that delay recovery and these include hypotension, urinary retention, pruritus, and motor blockade. Also, serious complications such as permanent nerve damage are rare, but remain a concern (Choi et al. 2003, Rawal 2012). Alternative means of postoperative analgesia are now more effective and commonly used after uncomplicated knee or hip replacement.

***Summary and recommendations****—Epidural analgesia is not recommended for routine use in hip and knee replacement because of the potential for adverse effects which delay recovery*

***Evidence level****—High (analgesic efficacy), moderate (negative safety and side-effect profile)*

***Recommendation grade****—Strong*

### Use of local anesthetics for nerve blocks and infiltration analgesia

Local infiltration analgesia (LIA) has an advantage over nerve blocks, because it produces no motor blockade. This can allow for earlier safe ambulation with a reduction in the side effects associated with both nerve blocks and epidural analgesia, where hypotension and urinary retention can cause additional problems.

Concerns with LIA have been raised regarding potential risks of local anesthetic toxicity, wound healing, and infection (McCartney and McLeod 2011). However, several reports have demonstrated that toxic levels do not appear to be reached using techniques described (Affas et al. 2011, Brydone et al. 2015). Several case series with long-term follow-up also demonstrate no increased risk of joint infection when using LIA compared with previous published data (Malviya et al. 2011, McDonald et al. 2012, 2016).

A review of the literature on LIA in total hip and knee replacement concludes that there is little evidence to support using this technique in hip replacement either intraoperatively or with a postoperative wound infusion catheter technique, as long as multimodal, oral non-opioid analgesia is given (Andersen and Kehlet 2014). For knee replacement, meta-analysis data support the intraoperative use of LIA (Yun et al. 2015), but not wound catheter administration postoperatively (Andersen and Kehlet 2014). There are no firm data suggesting one method of infiltration or a specific combination of drugs and dosage over another in LIA.

There are several nerve block techniques that may be used. Femoral nerve blockade has been compared and reviewed against epidurals and PCAs with advantages such as reducing the risk of hypotension and the use of opioids (Fowler et al. 2008, Paul et al. 2010, Chan et al. 2014). However, the main problem is its negative effect on mobilization (Kandasami et al. 2009, Ilfeld et al. 2010, Sharma et al. 2010). Whilst femoral nerve block is an effective analgesic technique following knee replacement, concerns remain regarding the impact on muscle function and early mobilization, with an increased risk of falls (Sharma et al. 2010).

The Hunter Canal block is an alternative to the femoral nerve block and is proposed to offer better preserved quadriceps muscle strength and mobilization ability in the 48 hours post-surgery (Jaeger et al. 2013, Perlas et al. 2013). However, conclusive data showing superiority has not been proven in established ERAS pathways.

The addition of a sciatic nerve block to the postoperative analgesic regimen following hip and knee replacement has not been found to provide substantial benefit (Paul et al. 2010) over any of the other alternative local techniques or the use of no local technique when assessed as part of a multimodal opioid-sparing analgesic regime.

***Summary and recommendations****—LIA is recommended for knee replacement but not for hip replacement within a multi-modal opioid sparing regime. Nerve block techniques provide equal analgesia; however, when compared with LIA prolonged motor blockade may limit early and safe mobilization. Nerve blocks are therefore not recommended as an essential ERAS component*

***Evidence level*** *for LIA in knee replacement—High*

***Recommendation grade****—Strong*

### Postoperative nausea and vomiting

Postoperative nausea and vomiting (PONV) is distressing for patients and can lead to notable patient morbidity and an associated prolonged length of hospital stay. In general, female gender, a past history of motion sickness or PONV, and being a non-smoker are all risk factors for PONV (Apfel et al. 1999). Several classes of first-line medications are available: dopamine (D2) antagonists (e.g., droperidol), serotonin (5HT3) antagonists (e.g., ondansetron), and corticosteroids (e.g., dexamethasone). Studies have shown that combinations of the drugs enhance their efficacy (Eberhart et al. 2002, Apfel et al. 2004). Therefore, in patients with 1 to 2 risk factors a combination of 2 drugs is often recommended, and, in patients with higher risk, three drugs in combination. If rescue treatment is required despite prophylaxis, drugs from classes not yet used should be employed (Gan et al. 2014).

***Summary and recommendation****—Evidence supports the use of screening for and multimodal PONV prophylaxis and treatment for patients undergoing hip and knee replacement*

***Evidence level****—Moderate*

***Recommendation grade****—Strong*

### Prevention of perioperative blood loss—tranexamic acid

Hip and knee replacement has been associated with pronounced blood loss (Liu et al. 2011), which, traditionally, has been healed by blood transfusion. However, its use carries risks including transfusion reaction, disease transmission, coagulopathy, renal failure, deep infection, and death (Juelsgaard et al. 2001). Studies are also required to evaluate the optimal transfusion triggers, especially in high-risk patients, where a lack of data has prevented inclusion in recent transfusion guidelines (Munoz et al. 2017, 2018). Historically, it has been shown to add to the cost of an operation and increase hospital stay (Newman et al. 1997). Decreasing blood loss and thus need for transfusion at the intraoperative stage may be achieved using combined local and systemic tranexamic acid, as this stops the breakdown of fibrin clot by inhibiting activation of plasminogen, plasmin, and tissue plasminogen activator. Studies have found it to be both efficient and safe (Rajesparan et al. 2009, Husted et al. 2010, 2014, Henry et al. 2011) despite previous concerns of an increased risk of venous thromboembolic events; and recent RCTs and meta-analyses support combined systemic and/or intra-articular administration (Nielsen et al. 2016, Shang et al. 2016, Zhang et al. 2017).

***Summary and recommendation****—Tranexamic acid is recommended to reduce perioperative blood loss and the requirement for postoperative allogenic blood transfusion*

***Evidence level****—High*

***Recommendation grade****—Strong*

### Multimodal analgesia

Combining oral analgesics of different classes and with different modes of action has been shown to yield additive pain relief (Ong et al. 2010). This is an effective way of limiting the use of opioids perioperatively and thereby avoiding their well-known side effects. In addition, prolonged opioid use in and after surgery is a leading risk factor for longer term addiction and so should be avoided (Clarke et al. 2014). The use of multimodal non-opioid oral analgesia is one of the cornerstones of exemplar ERAS hip and knee replacement pathways (McDonald et al. 2012, Khan et al. 2014). Paracetamol and non-steroidal anti-inflammatory drugs (NSAIDs) are the mainstay.

Optimal pain management is a prerequisite of ERAS and alternative analgesic drugs (such as glucocorticoids and ketamine) have been described for hip and knee replacement. Simple systemic techniques such as the addition of a high-dose preoperative glucocorticoid to multimodal regimes have been shown to be safe and effective in knee and hip replacement (Mathiesen et al. 2008, Lunn et al. 2011). However, there is a need for further data on dosage use in hip replacement patients and in at-risk groups such as high-pain responders.

### Paracetamol

Paracetamol is regularly prescribed perioperatively and within ERAS pathways. It reduces pain and morphine consumption over a 24-hour period in patients undergoing both hip and knee replacement (Sinatra et al. 2005). Although not specific to hip and knee replacement, paracetamol has been shown to reduce PONV if given prophylactically before surgery or arrival in the postoperative care unit and this correlated with a reduction in pain scores but not postoperative opioid use (Apfel et al. 2013). Paracetamol can reduce acute postoperative pain, has a favorable side-effect profile, and is a core component of multimodal analgesia in all exemplar hip and knee ERAS pathways.

***Summary and recommendations****—The routine use of paracetamol is recommended*

***Evidence level****—Moderate*

***Recommendation grade****—Strong*

### Non-steroidal anti-inflammatory drugs (NSAIDs)

Studies have shown that NSAIDs decrease pain and reduce supplemental analgesic (opioid) use following hip and knee replacement (Buvanendran et al. 2003, Huang et al. 2008). Level 1 evidence of postoperative analgesia following knee replacement determined that NSAIDs should be recommended for their analgesic and opioid-sparing effects (Fischer et al. 2008). NSAIDs are a central tenant of multimodal analgesia in exemplar hip and knee replacement ERAS pathways, and there is evidence from a randomized controlled trial that indicates no increase in perioperative blood loss, and accompanying reduction in pain (Meunier et al. 2007. However, individual patient risk should be assessed including the potential for bleeding complications, gastroduodenal ulcer history, cardiovascular morbidity, aspirin-sensitive asthma, and renal and hepatic function. Due to their side-effect profile, judicious use and appropriate patient selection is required. It should also be noted that the avoidance of NSAIDs postoperatively due to the risk of prosthetic loosening is an unsubstantiated fear that prevents patients from receiving evidence-based multimodal analgesia including NSAIDs (Husted et al. 2014). Additionally, it is important to avoid inappropriate use of NSAIDs in patients with pre-existing kidney disease (Bjerregaard et al. 2016b).

***Summary and recommendations****—The routine use of NSAIDS is recommended for patients without contraindications*

***Evidence level****—High*

***Recommendation grade****—Strong*

### Gabapentinoids

Several meta-analyses have suggested that gabapentinoids may reduce postoperative opioid consumption, pruritus, and nausea following total hip and knee replacement surgery, and improve sleep, and indeed gabapentinoids have been included in exemplar peer-reviewed ERAS cohorts (Malviya et al. 2011). However, there is a lack of evidence as to whether such drugs reduce pain (Lunn et al. 2015, Han et al. 2016a, 2016b, Hamilton et al. 2016, Zhai et al. 2016, Mao et al. 2016, Petersen et al. 2018b), and so gabapentinoids are not recommended.

***Summary and recommendation****—Gabapentinoids are not currently recommended as an adjunct in a multimodal analgesia regime although further studies are indicated*

***Evidence level****—Moderate (for not recommending)*

***Recommendation grade****—Strong*

### The use of supplemental opioid analgesia

ERAS programs emphasize the desire to minimize the use of opioids postoperatively and utilize alternative forms of analgesia. Nevertheless, their use when required is routinely reported in exemplar ERAS centers (Husted 2012). The efficacy of opioids in reducing pain scores after surgery is well established. However, concerns remain regarding side effects including drowsiness, respiratory depression, nausea and vomiting, pruritus, urinary retention, and potential risk of long-term addiction.

Currently, opioid analgesics are used if needed within the immediate postoperative period. They can be used to enable a smooth transition from peripheral techniques (e.g., LIA or PNBs) to non-opioid analgesia. The choice of opioid and method of delivery has been debated. Several studies investigated the use of controlled-release (CR) oxycodone following hip and knee replacement. Equivalency of analgesic effect has been demonstrated (Rothwell et al. 2011), and CR oxycodone has been associated with shorter hospital length of stay and better tolerance compared with PCA regimes (de Beer et al. 2005). Furthermore, by removing the required IV access and connection to a PCA pump, patients are more readily able to function independently (e.g., dress/shower/ambulate) and achieve the desired discharge criteria, which reduces the need for supervision to assist with movement of equipment that can adequately be replaced with oral medication. Therefore, it is strongly recommended that the use of such pumps is limited in the routine arthroplasty surgical population.

***Summary and recommendation****—ERAS programs seek to minimize the use of opioids. However, opioids such as oxycodone may be used when required as part of a multimodal approach*

***Evidence level****—High*

***Recommendation grade****—Strong*

### Maintaining normothermia

The National Institute for Clinical Excellence (NICE) recommends the pre-warming of patients and to maintain the active warming of all adults undergoing surgery throughout the intraoperative phase (NICE 2016). Multiple series suggest that normothermia should be targeted as part of the anesthetic care of hip and knee replacement patients. There are many methods described to conserve body temperature, including pre-warming and humidification of anesthetic gases, warming IV and irrigation fluids, and forced air-warming blankets and devices. However, the use of forced air-warming is not recommended as there is evidence that this is associated with an increased risk of infection (McGovern et al. 2011, Koc et al. 2017). In addition, the ambient temperature should be at least 21 °C while the patient is exposed prior to active warming starting (NICE 2016).

***Summary and recommendations****—Normal body temperature should be maintained peri- and postoperatively through pre-warming and the active warming of patients intraoperatively*

***Evidence level****—High*

***Recommendation grade****—Strong*

### Antimicrobial prophylaxis

Infection after hip and knee replacement is a serious complication that can be difficult to treat (Zimmerli et al. 2004). There is currently no universal internationally defined guideline for antibiotic/antiseptic prophylaxis for hip and knee replacement, with differing national and local policies in existence (Voigt et al. 2015). However, a recent consensus paper does present recommendations for type, timing, dosing, and repetition of antimicrobials (Aboltins et al. 2019). In a meta-analysis of total joint arthroplasty, antibiotic prophylaxis reduced the absolute risk of wound infection by 8% and the relative risk by 81% compared with no prophylaxis (p < 0.001). No other comparison showed a statistically significant difference in clinical effect (AlBuhairan et al. 2008).

Antibiotic-loaded bone cement may reduce infection rates after joint replacement. The evidence is more robust in hip compared with knee replacement (Engesaeteret al. 2006, Dale et al. 2009, Bohm et al. 2014). A recent systematic review and meta-analysis concluded that there is a paucity of well-conducted trials, and evidence of the protective effect is insufficient to recommend routine use in knee replacement (Hinarejos et al. 2015). Additional research into the role of antibiotic-loaded bone cement may also address concerns regarding patient safety, risk of antibiotic-resistant microorganisms, and increased cost.

***Summary and recommendations****—Patients should receive systemic antimicrobial prophylaxis in accordance with local policy and availability*

***Evidence level****—Moderate*

***Recommendation grade****—Strong*

### Antithrombotic prophylaxis treatment

Hip and knee replacement may be associated with deep venous thrombosis (DVT) and pulmonary embolism (PE), which can lead to post-thrombotic syndrome (PTS) or death (Husted et al. 2010). Evidence-based guidelines from the American College of Chest Physicians (ACCP) for DVT prophylaxis (Falck-Ytter et al. 2012) suggest a minimum of 10 to 14 days’ antithrombotic prophylactic use for patients undergoing hip and knee replacement. Comparatively, national guidelines, such as those made by NICE in the UK, advocate early mobilization, and the use of chemoprophylaxis for 28 days for hip replacement and 14 days for knee replacement postoperatively (NICE 2018). However, many of the data behind these recommendations are from older studies with traditional care pathways with less focus on early mobilization. More recent guidelines from the European Society of Anaesthesiology (Venclauskas et al. 2018) focus on day surgery and ERAS pathways, and take and provide recommendations for thromboprophylaxis in ambulatory or fast-track surgery derived from personal and procedure risk factors.

This tailored approach is consistent with the protocols presented in the large Danish observational studies of hip and knee replacement patients on ERAS pathways, where the incidence of thromboembolic events has been found to be substantially lower compared with historical figures. This has led to the abandonment of routine prophylaxis in favor of selective treatment in Denmark (Husted et al. 2010, Jorgensen et al. 2013, Jorgensen and Kehlet 2016). A subsequent recent large observational study on 17,582 patients has confirmed the safety of in-hospital only prophylaxis for those patients staying less than 5 days in hospital, and highlighted that further studies are needed to define optimal prophylaxis for high-risk patients and those who stay in hospital longer than 5 days (Petersen et al. 2018a).

***Summary and recommendations****—Patients should be mobilized as soon as possible post-surgery and receive antithrombotic prophylaxis treatment in accordance with local policy*

***Evidence level****—Moderate*

***Recommendation grade****—Strong*

### Perioperative surgical factors

#### Surgical technique

There is a multitude of literature reporting that the use of specific surgical techniques and approaches can accelerate the achievement of discharge criteria. Studies are conflicting and there is no strong evidence to illustrate the isolated effect of any one approach against another (Aggarwal et al. 2019, Jia et al. 2019). In a comprehensively reported and inclusive cohort series of ERAS outpatient hip and knee replacement surgery, all hip replacements were performed using a standard posterolateral approach with standard prosthesis, and all knee replacements were performed with a standard medial parapatellar approach with a standard prosthesis (Gromov et al. 2017). However, for example other studies on hip replacement have demonstrated success in same day discharge when adopting the direct anterior approach (Goyal et al. 2017, Berend et al. 2018), and conversely the posterior approach has been found to have a lower overall complication rate compared to the anterior approach (Aggarwal et al. 2019). Therefore, at present insufficient evidence exists to recommend that one surgical technique (type of approach, use of a minimally invasive technique, prosthesis choice, or use of computer navigation or robot) over another will independently effect achievement of discharge criteria within an ERAS set-up.

***Summary and recommendation****—There is no conclusive evidence that choice of surgical approach accelerates the achievement of discharge criteria. Therefore no recommendation can be given*

***Evidence level****—High*

***Recommendation grade****—Strong*

### Use of tourniquet for knee replacement

Tourniquet use in knee replacement is used with the aim to reduce bleeding. However, studies show that it does not reduce total blood loss and its use may cause swelling and impair early functional recovery (Li et al. 2009, Smith and Hing 2010). Studies have also found an increased risk of thrombosis and wound complication with tourniquet use, and no evidence of a better quality of cementation (Prasad et al. 2007, Husted et al. 2014, Zhang et al. 2014, Liu et al. 2017). No difference has been found in preserving knee-extension strength between surgeries with or without a tourniquet for patients on an ERAS pathway (Harsten et al. 2015a); although studies on standard care pathways have found differences in strength after surgery in favor of no tourniquet (Dennis et al. 2016, Guler et al. 2016) .

***Summary and recommendation****—The routine use of a tourniquet is not recommended*

***Evidence level****—Moderate*

***Recommendation grade****—Strong*

### Drainage

Level 1 evidence does not support the routine use of drains because they do not have any positive effect on the aims of their intended use such as for wound infections, hematomas, and healing complications (Parker et al. 2007, Quinn et al. 2015, Zhang et al. 2018). They have not been used in proven and well-documented hip and knee ERAS pathways (Husted et al. 2014), with no increase in complications, and their use may in fact increase blood loss and transfusion rate (Kelly et al. 2014).

***Summary and recommendation****—The routine use of surgical drains is not recommended for hip and knee replacement*

***Evidence level****—Moderate*

***Recommendation grade****—Strong*

### Perioperative fluid management

Recent advances in perioperative care have reduced the length of time that patients are nil by mouth prior to and following surgery. This has enabled rapid return of gastrointestinal function, particularly after major abdominal operations (NICE 2017). Maintaining fluid balance is, therefore, a prominent component of general surgical ERAS pathways. However, due to limited intraoperative blood and fluid loss and the early intake of postoperative oral fluids, the term “effective fluid management” remains ambiguous in lower limb primary replacement. We are aware of only 1 study on fluid management on an ERAS pathway for total knee replacement (Holte et al. 2007), which found that liberal fluid management, when compared with restrictive fluid management, may lead to hypercoagulability and a reduction in vomiting, with no differences found for postoperative hypoxemia, exercise capacity, recovery variables, or length of stay.

There have been limited investigation of intraoperative techniques such as goal directed fluid therapy in hip and knee replacement. However, large observational studies in ERAS hip and knee settings have highlighted that acute kidney injury is most often due to pre-existing kidney disease and postoperative hypotension, indicating that an increased focus on perioperative fluid management is important in the perioperative care of patients with pre-existing kidney disease (Bjerregaard et al. 2016b).

Care should be taken to detect and avoid electrolyte imbalance including hyponatremia (Sah 2014). Intravenous fluids should be judiciously used with the aim of providing routine maintenance fluids to meet insensible losses, to maintain normal status of body fluid compartments and enable renal excretion of waste products (NICE 2017). Routine maintenance provision should nearly always be a short-term measure and postoperative intravenous fluids discouraged in favor of early oral intake.

***Summary and recommendation****—It is recommended that intravenous fluids should be used judiciously and postoperative intravenous fluids discouraged in favor of early oral intake*

***Evidence level****—Moderate*

***Recommendation grade****—Strong*

### Urinary catheter use

Urinary catheters have been used routinely for longer surgical procedures to monitor urinary output and guide fluid resuscitation (Huang et al. 2015). However, their use has been questioned due to improved blood-saving and anesthetic practices. Studies have found no benefit in the use of an indwelling catheter when compared with no catheter or intermittent catheterization (Balderi and Carli 2010, Huang et al. 2015). Additionally, a low incidence of serious renal and urological complications has been found following hip and knee replacement surgery for patients on an ERAS pathway (Bjerregaard et al. 2016a). Therefore, the evidence indicates that the routine use of urinary catheters should be avoided. Postoperatively, a large RCT performed in an ERAS pathway has also demonstrated that a catheterization threshold of 80 0mL compared with 500 mL significantly reduced the need for postoperative urinary catheterization, without increasing urological complications (Bjerregaard et al. 2016b).

***Summary and recommendation****—The routine use of urinary catheters is not recommended and when used they should be removed as soon as the patient is able to void, ideally within 24 hours after completion of surgery. A catheterization threshold of 800 mL should be used to reduce the need for postoperative urinary catheterization*

***Evidence level****—Moderate*

***Recommendation grade****—Strong*

### Postoperative nutritional care

No studies have investigated the direct association of early feeding or postoperative nutritional supplementation with the accelerated achievement of discharge criteria. However, return to normal food intake is considered an essential component of ERAS protocols in order to achieve return to normal behaviors. Early return to normal diet is a central component of all exemplar ERAS pathways, with units encouraging patients to eat and drink as soon as they feel able.

***Summary and recommendation****—An early return to normal diet is recommended and should be promoted*

***Evidence level****—Low*

***Recommendation grade****—Strong*

### Early mobilization

Patients should be mobilized as soon as possible following surgery. Outpatient surgery is now established for 13–15% of unselected patients, and patients are routinely discharged on the first and second postoperative day (Gromov et al. 2017) establishing early mobilization as essential. This is supported by level 1 evidence that early mobilization reduces length of stay (Guerra et al. 2015). This counteracts the long-recognized adverse physiological effects associated with prolonged bed rest such as increased insulin resistance, muscle atrophy, reduced pulmonary function, impaired tissue oxygenation, and increased risk of thromboembolism (Harper and Lyles 1988).

***Summary and recommendation****—Patients should be mobilized as early as they are able to in order in order to facilitate early achievement of discharge criteria*

***Evidence level****—Strong*

***Recommendation grade****—Strong*

### Criteria-based discharge

A feature of exemplar hip and knee replacement ERAS pathways is that patients are discharged directly to their home and that objective discharge criteria are used. These criteria clearly define the requirements for going home from hospital, and are typified by including elements such as the ability to dress independently, the ability to get in and out of bed, the ability to sit and rise from a chair/toilet, the ability to be independent with personal care, and independent mobilization with walker/ crutches, and the ability to walk > 70 m with crutches (Husted et al. 2011, Scott et al. 2013).

***Summary and recommendation****—Objective discharge criteria should be used to facilitate patient discharge directly to their home*

***Evidence level****—Low*

***Recommendation grade****—Strong*

### Continuous improvement and audit

The continual review of clinical practice and outcomes is a critical component of ongoing quality improvement in healthcare. Experience from other ERAS procedures indicates that the relative effectiveness of audit and feedback is likely to be greater when baseline adherence to recommended practice is low (Gustafsson et al. 2011). Compliance with ERAS processes has been found to be lower than expected in other procedures, with large studies in colorectal surgery reporting compliance levels of around 60% (ERAS Compliance Group 2015). Therefore, an audit process that incorporates data collection, and the reviewing of one’s practices against ongoing gathering of evidence (performed either in-house or by others) is an important factor in ERAS pathways (Ljungqvist et al. 2017). Experience from other procedures indicates that the 4 main roles of an internal or external audit cycle are to: (1) measure clinical outcomes (such as length of stay, readmissions, and complications); (2) measure non-clinical outcomes (such as economics, and patient satisfaction/experience); (3) measure process compliance with ERAS components; and (4) maintain the concept as dynamically as possible (including new available evidence and modifying the multimodal concept).

***Summary and recommendation****—Routine internal and/or external audit of process measures, clinical outcomes, cost-effectiveness, patient satisfaction/experience, and changes to the pathway is recommended*

***Evidence level****—Low*

***Recommendation grade****—Strong*

## Comment

This document outlines the recommendations of the ERAS Society for the perioperative management of patients undergoing hip and knee replacement surgery, and summary details are provided in Table 3. It is based on the best available evidence as judged by these authors, which demonstrates that when using an ERAS pathway unselected patients can be routinely discharged from hospital 0–3 days following surgery, with no increased effect on morbidity or mortality (Aasvang et al. 2015, Gromov et al. 2017). These guidelines are an important document in summarizing the large volume of heterogeneous studies across all ERAS components within hip and knee replacement surgery.

**Table 3.** Summary of recommended interventions for the perioperative care of hip and knee replacement

Number Item	Recommendation	Evidence level	Recommendation grade	Number Item
1	Preoperative information,	Patients should routinely receive preoperative education education and counseling	Low	Strong
2	Preoperative optimization	4 weeks’ or more smoking cessation is recommended prior to surgery.	Smoking: High	Strong
		Alcohol cessation programs are recommended for alcohol abusers	Alcohol: Low	Strong
		Anemia should be actively identified, investigated, and corrected preoperatively	High	
3	Preoperative fasting	Clear fluids should be allowed up to 2 h and solids up to 6 h hours prior to induction of anesthesia	Moderate	Strong
4	Standard anesthetic protocol	General anesthesia and neuraxial techniques may both be used as part of multimodal anesthetic regimes	General anesthesia: moderate neuraxial techniques: Moderate	Strong
5	Use of local anesthetics for infiltration analgesia and nerve blocks	Within a multimodal opioid-sparing analgesic regimen, the routine use of LIA is recommended for knee replacement but not for hip replacement	LIA in knee replacement: High	Strong
		Nerve block techniques have not shown clinical superiority over LIA		
6	Postoperative nausea and vomiting	Patients should be screened for and given multimodal PONV prophylaxis and treatment	Moderate	Strong
7	Prevention of perioperative blood loss	Tranexamic acid is recommended to reduce perioperative blood loss and the requirement for postoperative allogenic blood transfusion	High	Strong
8	Perioperative oral analgesia	A multimodal opioid-sparing approach to analgesia should be adopted	Paracetamol: Moderate	Strong
		The routine use of paracetamol and NSAIDs is recommended for patients without contraindications	NSAIDS: High	Strong
9	Maintaining normothermia	Normal body temperature should be maintained peri- and postoperatively	High	Strong
10	Antimicrobial prophylaxis	Patients should receive systemic antimicrobial prophylaxis	Moderate	Strong
11	Antithrombotic prophylaxis treatment	Patients are at increased risk of VTE and should undergo pharmacologic and mechanical prophylaxis in line with local policy	Moderate	Strong
12	Perioperative surgical factors	There is no conclusive evidence that choice of surgical approach accelerates the achievement of discharge criteria	High	Strong
		Therefore no recommendation can be given		
13	Perioperative fluid management	Fluid balance should be maintained to avoid over- and under-hydration	Moderate	Strong
14	Postoperative nutritional care	An early return to normal diet should be promoted	Low	Strong
15	Early mobilization	Patients should be mobilized as early as they are able in order to facilitate early achievement of discharge criteria	Moderate	Strong
16	Criteria-based discharge	Team-based functional discharge criteria should be used to facilitate patient discharge directly to their home	Low	Strong
17	Continuous improvement and audit	Routine internal and/or external audit of process measures, clinical outcomes, cost effectiveness, patient satisfaction/experience, and changes to the pathway is recommended	Low	Strong

The aim is to provide a starting point for implementation for teams new to ERAS, and as a point of reflection for experienced ERAS teams to examine their current practice. These guidelines, and the testing of their implementation, as has been performed in other ERAS procedures, will hopefully allow us to consolidate consensus within the evidence base, and generate new evidence, through systematic prospective data collection and through clinical trials.

Future work should focus on reaching the goal of the “pain and risk free” hip and knee replacement. In order to do this, we will need to better understand the pathophysiological mechanisms of recovery, and the potential to optimize post-discharge functional outcomes (Wainwright and Kehlet 2019). This will be important because, for some of the ERAS components, there is a strong need for properly designed randomized controlled studies that are sufficiently powered, performed in ERAS settings, and that allow for discrimination between outcome parameters. With length of stay now reduced to between 0 and 3 days, post-discharge markers of recovery will become increasing important in order to discriminate interventions.

More specifically, work is still required in order to understand how to reduce the inflammatory response postoperatively; how to further reduce pain; how to reduce impairment of physical activity and improve function quicker postoperatively; how to better identify patients at high risk of complications owing to psychiatric disorders, chronic renal failure, and orthostatic intolerance; anemia and transfusion thresholds; postoperative urine retention and urinary bladder catheterization; and how to improve sleep (Wainwright and Kehlet 2019). Intertwined with this will be the need for further research on the feasibility of same-day surgery, and the type (e.g., exercise therapy, cryotherapy, electro-neuromuscular stimulation), timing and duration of physiotherapy post-discharge (Bandholm et al. 2018, Wainwright and Kehlet 2019).

## Conflicts of interest

TW—Consulting contracts with: Medtronic, ZimmerBiomet. Department/research funding from: ZimmerBiomet, Stryker, Lima Corporate.

MG—No relevant conflicts of interest.

DM—No relevant conflicts of interest.

RM—Consulting contracts with: Stryker, ZimmerBiomet. Department/research funding from: ZimmerBiomet, Stryker, Lima Corporate.

MR—Speakers’ Bureau: Heraeus, ZimmerBiomet, Stryker. Research funding: Heraeus Medical GMHB, ZimmerBiomet, 3M, Curetis GMHB, Vifor Pharma, Schuelke. Other financial/material support: Stryker, ZimmerBiomet, Heraeus, Biocomposites, Aquilant, Depuy, Smith & Nephew, Bone and Joint Infection Registry.

OS—No relevant conflicts of interest.

PY—Consulting contracts with: DepuySynthes, Zimmer, Global, Palacos, Corin, Matortho. Designer for: MatOrtho, Global Orthopaedics, Zimmer, Synthes, Corin. Department/research funding from: DepuySynthes, Zimmer, Global, Smith & Nephew, Stryker, Matortho.

OL—Co-founder of and Chairman of the Executive Committee of the ERAS Society. Founder and shareholder in Encare AB, Advisor to Nutricia, NL; Advanced Medical Nutrition, Can. Speaker’s honoraria and travel support from Nutricia, Fresenius-kabi, BBraun, Merck, Medtronic, and Baxter.
